# A comprehensive literature-based equation to compare cost-effectiveness of a flexible ureteroscopy program with single-use versus reusable devices

**DOI:** 10.1590/S1677-5538.IBJU.2018.0880

**Published:** 2019-09-02

**Authors:** Giovanni S. Marchini, Fábio C. Torricelli, Carlos A. Batagello, Manoj Monga, Fábio C. Vicentini, Alexandre Danilovic, Miguel Srougi, William C. Nahas, Eduardo Mazzucchi

**Affiliations:** 1 Seção de Endourologia da Divisão de Urologia do Hospital das Clínicas da Faculdade de Medicina da Universidade de São Paulo, SP, Brasil;; 2 Glickman Urological and Kidney Institute, Cleveland Clinic, Cleveland, Ohio, United States

**Keywords:** Cost-Benefit Analysis, Ureteroscopy, Kidney Calculi

## Abstract

**Purpose:**

to critically review all literature concerning the cost-effectiveness of flexible ureteroscopy comparing single-use with reusable scopes.

**Materials and Methods:**

A systematic online literature review was performed in PubMed, Embase and Google Scholar databases. All factors potentially affecting surgical costs or clinical outcomes were considered. Prospective assessments, case control and case series studies were included.

**Results:**

741 studies were found. Of those, 18 were duplicated and 77 were not related to urology procedures. Of the remaining 646 studies, 59 were considered of relevance and selected for further analysis. Stone free and complication rates were similar between single-use and reusable scopes. Operative time was in average 20% shorter with digital scopes, single-use or not. Reusable digital scopes seem to last longer than optic ones, though scope longevity is very variable worldwide. New scopes usually last four times more than refurbished ones and single-use ureterorenoscopes have good resilience throughout long cases. Longer scope longevity is achieved with Cidex and if a dedicated nurse takes care of the sterilization process. The main surgical factors that negatively impact device longevity are lower pole pathologies, large stone burden and non-use of a ureteral access sheath. We have built a comprehensive financial cost-effective decision model to flexible ureteroscope acquisition.

**Conclusions:**

The cost-effectiveness of a flexible ureteroscopy program is dependent of several aspects. We have developed a equation to allow a literature-based and adaptable decision model to every interested stakeholder. Disposable devices are already a reality and will progressively become the standard as manufacturing price falls.

## INTRODUCTION

Flexible ureterorenoscopes are expensive to acquire and have limited longevity (1). The significant improvements in flexible ureterorenoscopes have made flexible ureteroscopy the main treatment modality to target upper urinary pathologies, especially stone disease (2-4). The low invasiveness of the procedure has made it popular worldwide. Nevertheless, there is growing concern globally regarding its high costs (5). Also, one must consider the costs of the laser machine used for stone fragmentation, personnel to take care of cleaning and sterilization processes, and all the disposable instruments used within the flexible ureteroscope procedure which have made it so efficient. Finally, when a reusable scope breaks, some institutions may experience a significant delay for its replacement or repair, obligating to have more than one device so that the surgical program is not suddenly interrupted (3-5).

On the other hand, we are now entering in the era of single-use devices (6). In principle, the disposable ureterorenoscope eliminates the high costs of reusable scopes purchase and repair. It also abolishes the theoretical risk of cross infections and the need for a sterilization process. Additionally, some advocate that the disposable scope allows more torque in the instrument during a stone treatment procedure without the fear of breakage, pushing flexible ureteroscopy boundaries further.

The purpose of this study was to critically evaluate all studies concerning the cost-effectiveness of flexible ureteroscopy comparing single-use with reusable scopes in order to create a comprehensive equation to allow a literature-based decision.

## MATERIALS AND METHODS

A systematic online literature review was performed in PubMed, Embase and Google Scholar databases. The following key words were used to attain relevant studies regarding flexible ureteroscopy using reusable and disposable scopes: “flexible” combined with the terms “ureteroscopy”, “ureteroscope”, “ureterorenoscopy”, “ureteroscopic”, “ureteropyeloscopy”, “durability”, “longevity”, “cost-analysis”, “digital”, “fiber-optic”, “single-use”, “disposable”, “reusable”, “renal”, “urinary” and “sterilization”.

We performed the review of all published studies on flexible ureteroscopy in order to establish a literature-based decision model for flexible ureteroscope acquisition. For that, we aimed to answer pre-defined questions formulated by two experienced endourologists (GSM and FCT) who work on private and public institutions with different medical reimbursement policies and distinguished surgical supplies used for endourological procedures. These queries were designed to evaluate the clinical and economic impact of the type of flexible ureteroscope used on daily practice and are the following:

1. Are the stone free and complication rates different between single-use and reusable flexible ureteroscopes?
2. Is the operative time different between single-use and reusable flexible ureteroscopes?
3. Does surgeon experience impact clinical and economic outcomes in a individualized manner between single-use and reusable scopes?
4. Is the longevity of digital and optical flexible ureteroscopes different? Also, is it different between new and refurbished scopes?
5. Does the sterilization method influence permanent scope longevity?
6. What is the impact of stone burden and surgical instrumentation on ureteroscope longevity?
7. How may we generate a cost-analysis equation based on the above-mentioned criteria to allow an objective and literature-based decision model to elect the most suitable flexible ureteroscope acquisition policy for our institution?

Our procedure for evaluating records identified during the literature search followed the Preferred Reporting Items for Systematic Reviews and Meta-analyses (PRISMA) criteria (7). All the relevant studies were gathered, organized, and brought to discussion. Two separate urologists performed the online search and reviewed all papers considered suitable and relevant for this analysis. Because of the paucity of high-quality publications, not only prospective assessments but also case control and case series studies were included in the final analysis.

## RESULTS

**Figure 1:**
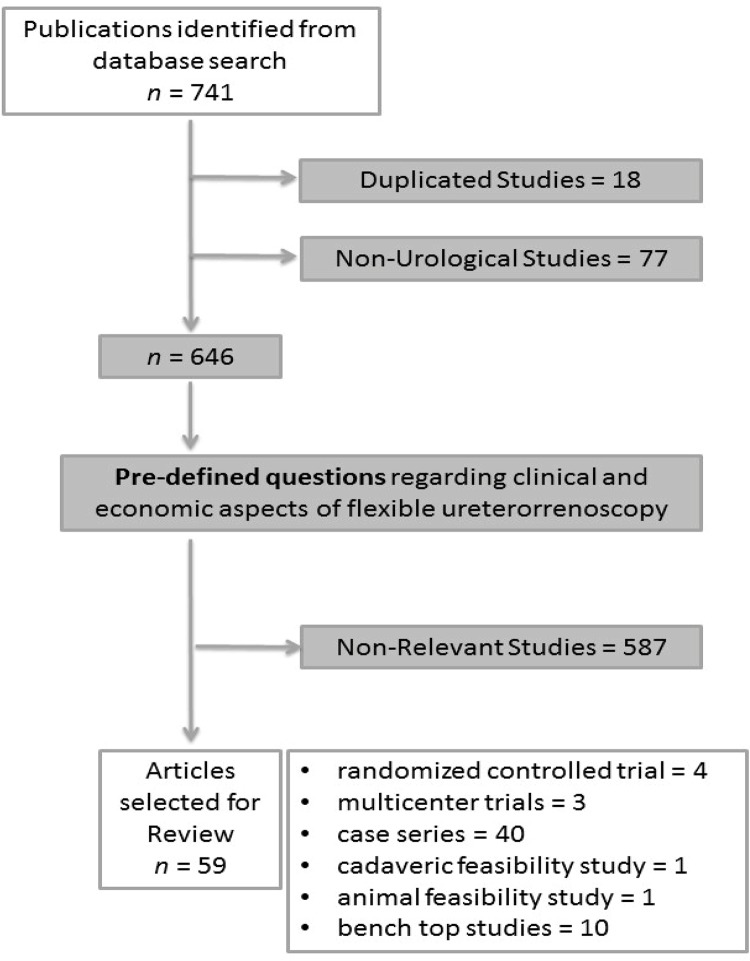
Flow diagram of evidence acquisition in a systematic review on single-use and reusable flexible ureteroscopy.

After extensive review of the literature, 741 studies with the previously elected terms were found (Figure-1). Of those, 18 were duplicated and 77 were not related to urology procedures and were excluded. Of the remaining 646 studies, 59 published between 2000 and 2018 were considered of relevance. The studies details are exposed on Table-1. The experts who carefully selected all studies formulated literature-based answers to the articulated questions and developed a comprehensive decision-model equation for maintaining a flexible ureteroscopy program.

**Table 1:** – Study details regarding design and type of flexible ureteroscope used.

Study Design	References
Randomized controlled trials (Level evidence 1)	9,11,38,56
Multicenter trials (Level evidence 4)	17,30,33
Case series (Level evidence 4)	5,8,10,12,13,15,16,25-29,31,32,34-37,39,41,42, 44-52,54,55, 57-63,66
Cadaveric feasibility study (Level evidence 4)	18
Animal feasibility study (Level evidence 4)	19
Bench top studies (Level evidence 4)	14,20-24,40,53,64,65
**Flexible Ureteroscope Included**	**References**
Olympus URF-P3	14,26,30,39,43,48, 55,56,62,66
Olympus URF-P5	5,9,18,19,33,44,46,54,61
Olympus URF-P6	10,13,49
Karl Storz Flex-X	14,30-32,39-41,50,59
Karl Storz Flex-X^2^	11,28,35,45,47,57,58,60,61
ACMI DUR-8 / DUR-8 elite	14,29,30,33,36,37,39,59
Richard Wolf 7330/1	14,26,30,39,63,64
Richard Wolf Cobra	20,24
Richard Wolf Viper	33
ACMI AUR-7	26
Stryker Flex Vision U-500	33
Olympus URF-V	5,18,27,28,51,52
ACMI/Olympus DUR-D	38
Karl Storz Flex-X^C^	11,20,24,25,28,35,42
LithoVue^TM^	10,11,13,20,22-24,42,51,52
Polyscope^TM^	8,9,15,16,21,34
SemiFlex^TM^	14
Pusen^TM^	23
YouCare Tech YC-FR-A^TM^	24
Neoscope NeoFlex^TM^	24

### Stone Free and Complication Rates

Somani et al. published a study comparing reusable digital versus fiber optic flexible ureteroscopes and the results were similar in terms of accessibility to the entire collecting system and stone-free rates (SFRs). Complication rates were similar between the two modalities (5). Nevertheless, the authors did not use disposable scopes.

The Polyscope^TM^ has been introduced in urologic armamentarium as a modular, semi disposable flexible ureterorenoscope system (8). One study prospectively compared clinical outcomes of the Polyscope^TM^ with reusable Olympus fiberoptic URF-P5 scope (9). After including 180 patients in each arm, the single session SFR postoperatively for Polyscope^TM^ and URF-P5 was 76.7% versus 69.4% (p=0.12), respectively. However, for lower calyceal stones, URF-P5 was significantly better than Polyscope^TM^ (82.0% vs. 69.2%; p=0.022), respectively. The complication rate was 15.3% versus 15% (p=0.3), respectively. Urosepsis occurred in 5% of patients in the Polyscope^TM^ group and 3.3% in the reusable scope cohort (p=0.42).

Usawachintachit et al. performed a more recent prospective case-control study in which LithoVue^TM^ was compared to Olympus fiberoptic URF-P6 (10). A total of 116 cases were performed with single-use scope and 65 cases with reusable scopes. The number of patients with no fragments, insignificant residual fragments (≤2mm) and significant fragments (>2mm) was 60.0%, 12.5%, 27.5% for LithoVue^TM^, and 44.7%, 13.2%, 42.1% for URF-P6 (p=0.36), with a tendency towards better outcomes with the single-use scope. Mager et al. (11) prospectively compared 68 consecutive procedures using reusable flexible ureterorenoscopes (Flex-X2^S^/Flex-X^C^, Karl Storz) with 68 consecutive procedures utilizing single-use digital flexible ureterorenoscopes (LithoVue™). Patients had same stone burden and demographic characteristics. The authors found non-significant different SFR (82% vs. 85%; p=0.8) and complication rates (7 vs. 17%; p=0.06) with reusable and single use scopes, respectively. One febrile urinary tract infection (UTI) occurred in the single-use group and none in the reusable scope cohort.

Regarding perioperative complications, UTI remains a feared hurdle following ureteroscopy. A study in France reports that acute pyelonephritis is a rare complication of ureteroscopy (2.4%) (12). In the study by Usawachintachit et al. (10), the complication rate was lower in the LithoVue^TM^ group compared to the URF-P6 group (5.4% vs. 18.0%; p <0.05). Interestingly, there were three cases of UTI in each arm. Similar rates of UTIs were seen in other studies comparing single-use with reusable scopes (9, 11).

As the last generation single-use devices have shown similar characteristics to reusable ureterorenoscopes, allowing similar SFRs, the price analysis may be performed with the knowledge that differences in cost do not translate in surgical outcomes inconsistency. In the same sense, a cost reduction, no matter towards single-use or reusable scopes, will not impact morbidity.

### Operative Time

One study prospectively comparing the Polyscope^TM^ single-use flexible ureteroscope with reusable scopes found similar mean procedure duration: 73±27 versus 74±13min (p=0.99), respectively (9). In this study, both types of flexible ureteroscopes, disposable and reusable, were fiberoptic.

In the study by Somani and coworkers (5), the authors found that the digital flexible scope allowed a decreased operative time by 20% with similar SFR. In the study by Usawachintachit et al. (10), the overall mean procedure duration was 10.4 minutes shorter for LithoVue^TM^ than with fiberoptic URF-P6 (64.5 vs. 54.1min;p <0.05). This difference broadened to 13 minutes and remained statistically significant in cases performed for stone removal (70.3 vs. 57.3min;p <0.05). This was translated in shortened operating room duration in stone removal cases with LithoVue^TM^ (104.3 vs. 89.8min, respectively; p <0.05).

In a subsequent study from the same group, Tagushi et al. (13) prospectively compared flexible ureteroscopy with the fiberoptic Olympus URF-P6 and LithoVue^TM^ in a micro-cost analysis. They found a non-significant 19.8 min (93.4 vs. 73.6min; p=0.09) or 21% shorter total operative time with the digital single-use scope. This was translated in a mean reduction from US$ 1.618.72 to US$ 1.348.64 per procedure. In the study by Mager et al. (11), the author compared reusable scopes (Flex-X2^S^/Flex-X^C^, Karl Storz) with single-use LithoVue^TM^ and found similar operative time (76.2 vs. 76.8min; p=0.9). However the authors did not compare the specific operative time of single-use versus digital (Flex-X^C^) and fiberoptic (Flex-X2^S^) reusable scopes.

By analyzing the above-mentioned data, the final equation for calculating cost of a flexible ureteroscopy program should include an operative time factor and a higher effectiveness is achieved with digital scopes.

### Surgical Expertise Impact on Ureteroscope Efficiency

Several studies have evaluated the mechanical, optical and irrigation properties of single-use ureterorenoscopes (8, 9, 14-24). The more recent single-use scopes have good performance and do not lack in endurance and maneuverability compared to permanent equipment. A recent multi-institutional, prospective, comparative study by Usawachintachit et al. paralleled procedural outcomes between LithoVue^TM^ and reusable ureteroscopes (10). The authors found that the LithoVue^TM^ was associated with a shorter learning curve and had comparable procedural outcomes and complication rates when matched with reusable flexible optical ureteroscopes. Nevertheless, no dedicated learning-curve investigation was performed. The fact that a digital scope was compared to a fiberoptic one might explain the results.

There are no studies comparing scope longevity regarding single surgeon versus multiple surgeon’s use. However, a recent case series of flexible ureteroscopy using the Storz digital Flex-X^C^ by a single expert urologist with more than 1000 flexible ureteroscopies performed has shown long scope longevity with a single scope lasting 159 cases (25). It is common sense that inexperienced surgeons have a higher chance of damaging the flexible ureterorrenoscope if not properly supervised. As all surgical procedures, there is a learning curve that must be respected. Unfortunately, no study was able to identify the learning curve effect on single-use versus reusable scopes and this could not be included in the final equation for calculating the cost of a flexible ureteroscopy program.

### Longevity of digital, optical, new and refurbished flexible ureteroscopes

In average, a digital ureteroscope is used 21 times before requiring repair, while the average fiberoptic ureteroscope is only used 6-15 times before going back to the manufacturer (26, 27). In a recent study by Legemate et al. (28), reusable digital scopes had a slightly longer longevity (mean 27 cases; 20-56) compared to fiber optic flexible ureteroscopes (mean 24 cases;10-37). However, a wider look at all published literature reveal that new flexible scopes may last 5 to 159 cases (25-52). In comparison, the average longevity of refurbished flexible scopes ranges from 3 to 11 cases (35-39). In addition, one study suggests that not only brand-new flexible ureteroscopes are more resistant to damage (mean of 44 usages in this specific trial) than devices refurbished, but that scopes last more if they are repaired by original manufacturer (mean 11.1) than by outsourced vendors (mean 6.9 cases) (36).

In modern series with single-use flexible scopes, the resilience of the equipment was proved to be adequate even for long cases (9, 10, 13, 16). In the European prospective multicentric clinical study by Doizi et al. (17), however, there were two failures with LithoVue^TM^ (5%), which demanded the surgeons to use the permanent scope to finish the case. Scope longevity impacts the number of repair orders and was included in the final equation.

### Sterilization method impact on scope longevity

Different series investigating flexible ureteroscope breakage report that it may occur outside of the operating room, during processing and storage in 7.7 to 22% of times, even in the hands of experienced and dedicated staff (26, 29).

Abraham et al. (40) studied two identical fiber optic ureteroscopes that underwent two different sterilization processes: Steris 1 (peroxyacetic acid 35%; 30min cycle at 50°-56°C) and Cidex OPA (Johnson and Johnson Co., Irvine, CA; glutaraldehyde 2.4%; 30-40 min. soak cycle at room temperature followed by a rinse in sterile water). The authors have demonstrated that after 100 cycles, the first ureteroscope, which was sterilized in the Steris system, had a 12mm tear on its shaft, 297 damaged fibers, and a 37% drop in resolution. Conversely, the second ureteroscope, which was sterilized with Cidex, had no visible external damage and had only 10 damaged fibers.

In a clinical trial by McDougall et al. (48), a new Olympus URF-P3 flexible ureteroscope was used for two 30-day independent study periods during which a single surgeon used the endoscope for a variety of upper urinary tract procedures. During the first 30-day period (11 cases; operative time of 457 min.), the endoscope was cleaned by the endourology support team using the Steris 20. During the second 30-day period (15 cases; 618 min.), a separate endoscope was cleaned only by the surgeon using the Cidex technique. In follow-up evaluation of the flexible ureteroscopes, there was no change in the angle of flexion or deflection in either group during the study period and leak-proof-pressure testing was acceptable in both endoscopes. In Steris group, no optical fibers were noted to break during use. In Cidex group, during the study, eight fibers were broken. These findings are in discordance with the study by Abraham et al. (40). Still, the authors believe this was specifically related to a higher prevalence of lower pole stone location in the Cidex cohort and not to the sterilization process itself.

When we look at scope longevity, series that report longer scope duration are in general those where they were sterilized on Cidex and not Steris. Carey et al. report new scope duration of at least 48 cases using Cidex method (29). Delfidio et al. report fiberoptic ureteroscope duration of more than 100 cases for two scopes with the same process (32). Multescu et al. achieved the noteworthy mark of 159 procedures with a single digital Storz Flex-X^C^ (25). On the other hand, in a recent series by Mager et al. where Steris was the sterilization method (11), in 68 procedures utilizing reusable flexible ureterorenoscopes (Flex-X^C^ and Flex-X2^S^), 9 repair orders were needed caused by 5 damages of brand new and 4 damages of used instruments. In the study by Semins et al. (45), after all urology nurses had been educated by the charge nurse of the urology service as to the proper endoscope cleaning, processing, and sterilizing protocols with Steris system, the average number of uses per ureteroscope before repair was necessary increased from 10.8 to 28.1, with a repair cost saving of US$ 300.00 per use.

Single-use scopes have the clear advantage of not requiring any sterilization process as they are discarded at the end of the procedure. Only a minimal recycling cost might be considered. This translates in having a new scope for every procedure, theoretical lower risk of cross infection, and less cost related to reprocessing, logistics and personnel required for the whole cycle of scope sterilization. The final formula for calculating costs of the flexible ureteroscopy program contains a specific factor of reprocessing or recycling cost per case.

### Impact of stone burden and instrumentation on ureteroscope longevity

Several surgical and patient factors might affect stone free rates, morbidity and ureteroscope longevity (45). Access sheaths have been shown to protect the kidney and the ureter during flexible ureteroscopy and to potentially increase SFR (53). A large retrospective cohort confirmed the safety of the ureteral access sheath but failed to show any improvement in the stone free status among patients with compared to those in which the access sheath was not used (54). Pietrow et al. have reported that the routine use of ureteral access sheaths, miniaturized nitinol baskets and smaller laser fibers will minimize the strain placed on a ureteroscope during a procedure, ultimately increasing the flexible ureteroscope longevity (55). Other investigators have also suggested that the routine use of a ureteral access sheath may also help to improve the durability of the flexible ureteroscope since it provides continuous ureteral access, reduced ureteral trauma, and shorter operative times (56). Multescu et al. advocate routine use of an ureteral access sheath and have recently published a case series using three new generation digital flexible ureteroscopes in which they lasted for 96, 151 and 159 cases (25). However, to date, there are no well-designed prospective randomized trials to provide strong evidence that the durability of the deflection unit of the flexible ureteroscope is preserved using this technique.

Jacquemet et al. (57) retrospectively compared the outcomes of flexible ureteroscopy for stone treatment in patients with calculi in the lower pole (n=232) versus with calculi in other kidney locations (n=139). Stone burden was similar between groups but stone size <10mm (61.2% vs. 48.5%; p=0.018) and use of an access sheath were more frequent in the lower pole cohort (80.2% vs. 66.9%; p=0.007). In only 19.8% of these cases the calculus was relocated to a more favorable position in the kidney. SFR was similar between groups (68.3% in lower pole group vs. 69.8%; p=0.77) with no difference in regards to complication rates (9.1% vs. 7.9%, respectively; p=0.67). Jessen et al. retrospectively evaluated the influence of the collecting system anatomy on the efficacy and morbidity of flexible ureteroscopy and found that stone size, long infundibulum, and infundibulopelvic angle <30^o^ negatively affected the SFR (58). Perlmutter et al. retrospectively evaluated the impact of stone location on 86 cases managed by flexible ureteroscopy and laser lithotripsy and also found that stone location did not significantly affect the SFR (59). Martin et al. retrospectively compared 89 cases of flexible ureteroscopy for lower pole stones with 73 cases with stones in other locations and on multivariate analysis the presence of multiple stones was the only statistically significant predictive factor of SFR (60). Similar findings were reported by Resorlu et al., who pointed as independent factors for success the stone size, number, composition, infundibulopelvic angle and renal malformations (61). The common intraoperative practice of stone displacement with a basket or grasper into the renal pelvis or upper pole for lithotripsy could explain these findings (62, 63).

Although most studies support that SFR are similar for lower pole and non-lower pole stone, there is increasing evidence that there is a correlation between the technical difficulty of the procedure and a higher incidence of ureteroscope malfunction (64, 65). Auge et al reported that in situ fragmentation of lower pole calculi is not possible in 28–34% of cases because of reduced ureteroscope deflection caused by the optical fiber (66). In those cases, potential harm to the scope occurs. In forced deflections where the laser fiber is unable to maintain total internal reflection, the photons may refract into the cladding and jacket rather than reflect back into the core fiber, with resultant fiber failure and ureteroscope damage (30, 31). Forbes et al. retrospectively analyzed laser fiber logs during flexible ureteroscopy for stone treatment and found that malfunction occurred in 8 of 142 cases [5.6%] (50). Importantly, all 8 cases were in procedures for lower pole stones (8 of 79; 10.1%) and resulted in flexible ureteroscope damage. The combination of aggressive active deflection of the flexible ureteroscope and simultaneous passage of the holmium laser probe may stress the fiberoptic system and result in fiber breakage. In a recent series by Ozimek et al., the authors evaluated their reusable flexible ureterorenoscopy program and found that in 32 of 423 (7.5%) cases the scopes were defective after the procedures (51). Thirty-one of 32 cases (96.86%) with proven scope damage were related to exploration of the lower pole and in 20 of 23 (86.96%) it was for stone treatment in that location. Hennessey et al. treated 234 patients for renal stone procedures with seven new Olympus URF-V instruments and had 15 major scope damages in a 30-month period (52). Staghorn stones (p=0.016) and stones in the lower pole calyx or mid zone calyx (p=0.074) were risk factors for scope damage.

Stone burden and instrumentation affect scope longevity and were considered in the final equation for computing flexible ureteroscopy program costs.

### Cost-analysis decision model: creating a literature-based equation

The overall cost of a reusable scope must consider the financial expenditure for three main parameters: scope purchase, repair and sterilization. A recent series reported the cost of a new conventional flexible ureteroscope (Flex-X, Karl Storz, Germany) to be US$ 13.611 (41). The digital Olympus URF-V has been recently purchased by US$ 20.200 in an Australian series (52). The repair cost, diluted by case and scope longevity, also has a wide range in the literature from US$ 48 to US$ 605 per case (40-47, 51, 52). Both purchase and repair costs may be influenced by the business contract between the owner of the scope and the manufacturer or its dealer. The reprocessing or sterilization cost includes personnel (nurses, technicians), material for brushing, leakage testing, cleaning, packaging, and sterilization. If we do not consider the value of acquisition of STERRAD machine (system that use low-temperature, hydrogen peroxide gas plasma technology), recent cost-analysis studies show a reprocessing cost varying from US$ 19.9 to US$ 108.00 per case (11, 41, 49, 52).

When a disposable scope is being considered, repair should not be considered in the equation. Furthermore, there is no reprocessing, and this should be exchanged for recycling and labor. Tagushi et al. have shown a US$ 3.65 recycling cost per scope used (13). The main factor being considered for the single-use scope is always the purchase cost. This is mainly influenced by the generation of the disposable scope and by the business contract with the manufacturer. Recent purchase prices reported for the existing scopes are US$ 1300 to US$ 3180 for LithoVue^TM^ (22, 42, 52, 53), US$ 700 for Polyscope^TM^ (34), and US$ 800 for SemiFlex^TM^ (14). As the manufacturing process of single-use scopes become more effective, less instruments are discarded and final retail cost may fall. Furthermore, selling price also decreases as more brands are competing for the market share.

A recent investigation by Martin et al. (42) assessed the economic consequences of reusable flexible ureteroscopes by performing a cost-benefit analysis on all flexible ureteroscopies. Permanent digital Flex-X^C^ ureteroscopes were used in a total of 160 cases performed over a one-year period in which eight reusable scopes required repair. By using market price of LithoVue^TM^, the authors linearly extrapolated the single cost to the amount of cases in order to estimate the total expenditure of their flexible ureteroscopy program if a single-use device was used. They have demonstrated a cost of US$ 848.10 per case and favored reusable ureteroscopes only after 99 procedures were performed. The authors finally suggested that high-volume institutions might find reusable ureteroscopes more cost beneficial. Mager et al. (11) also made a cost-analysis study and found that cost of reusable flexible ureterorenoscopy ranged between US$ 436 and US$ 708 per case. When taking into consideration the initial purchasing costs, it increased to US$ 1212-US$ 1743 per case. In their series, LithoVue^TM^ had a price range of US$ 1300 (market price) to US$ 3180 (manufacturer’s suggested retail price) per procedure. In a prediction model, after 61 to 118 cases the routine use of a disposable scope would become more expensive than the routine use of reusable scopes.

In the German case series by Ozimek et al., the authors performed a retrospective evaluation of 102 diagnostic flexible ureteroscopies and 321 procedures for kidney stone treatment (51). The average number of cases resulting in scope damage was estimated to be 14.4 and the total cost of all procedures was estimated to be US$ 261.332. This resulted in an average cost per flexible ureteroscopy procedure of US$ 617.4 and the authors concluded that the reusable scope program was more cost-effective than if single-use scopes were employed since the assumed price per LithoVue^TM^ device was US$ 1.227.5.

**Figure 2:**
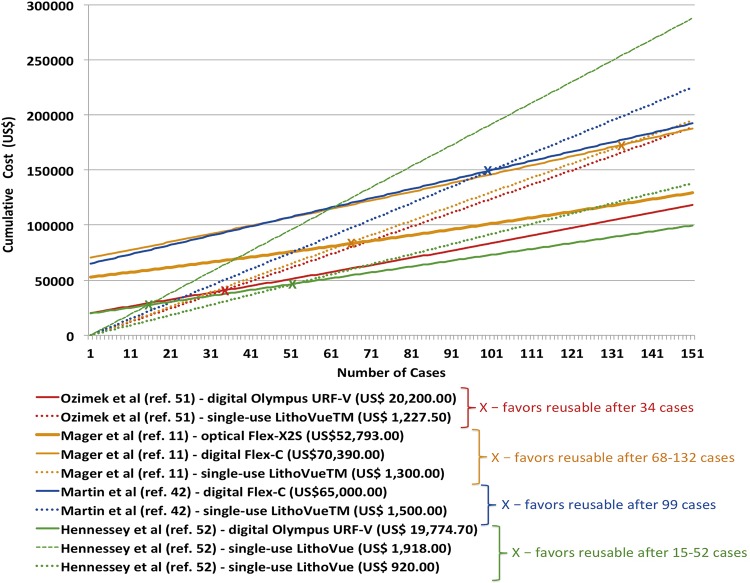
Cumulative cost analysis between single-use and reusable flexible ureteroscopes among different published series.

Hennessey et al. found a total repair cost for the 7 new digital scopes over the 30-month time period to be US$ 124.800, with a mean cost per case of US$ 533 (US$ 276-US$ 904) (52). The cumulative cost of 28 cases for the reusable flexible scope was approximately US$ 38.360. If the single-use scope (LithoVue^TM^) was priced at US$ 1.918, then it would cost approximately US$ 55.239 for the same 28 cases and reusable scopes would be more economical. Conversely, if the single-use disposable scope was priced at US$ 920, then the cost for 28 cases would be around US$ 26.850 and this would represent a considerable economical saving. Figure-2 depicts the economic projection of published data mentioned above, which did not account for operative time costs.

**Figure 3:**
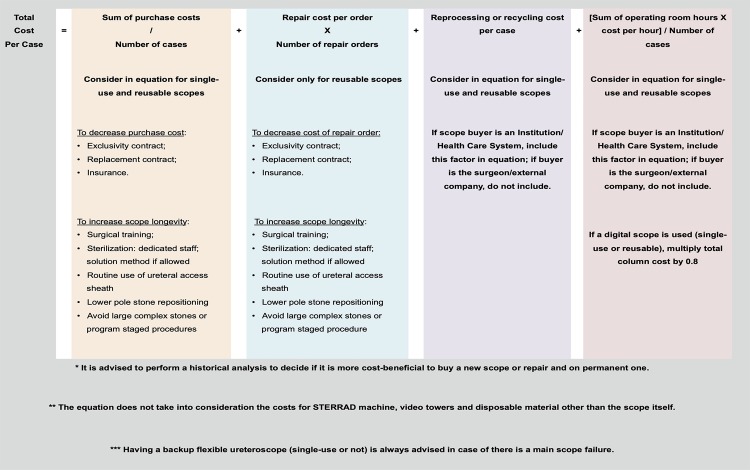
Literature-based equation to allow a cost-analysis decision model to flexible ureteroscope acquisition.

After extensive review of literature, the authors of this study used all information gathered as answers to the pre-defined questions to build a comprehensive literature-based equation to allow a financial cost-effective decision model to flexible ureteroscope acquisition (Figure-3). The main drivers in the analysis are purchase and repair costs when a reusable scope is being considered. That changes to purchase and recycling cost if the potential buyer aims a single-use scope. In addition, if an Institution or Health Care System is acquiring the flexible ureteroscope, one should also consider in the equation the reprocessing cost for reusable scopes and operating room cost for any scope type. Reprocessing costs and operating room cost may be omitted in the equation if the potential buyer is the surgeon or an external company since their expenditures are not influenced by those factors. Finally, if the operating room time is being considered, digital scopes allow performing the same lithotripsy procedure with 20% less time. Therefore, the cost decreases and a 0.8 factor should be considered in this specific portion of the equation.

## DISCUSSION

The first generation of disposable scopes had been tested and suboptimal surgical outcomes precluded their incorporation on daily practice (9, 15, 16). Newer scopes provide similar maneuverability and clinical efficacy to reusable scopes with equal low complication rates and are now part of the urology routine worldwide (10, 14, 17, 18, 20, 22-24, 42). Scope size seems not to be a significant issue for modern single-use digital scopes in comparison to reusable ureterorrenoscopes (10, 17, 18, 23). The trial by Usawachintachit et al. could even have shown superiority with LithoVue^TM^ compared to a reusable scope if a larger sample size was included in the study (10).

Higher complication rates translate into prolonged hospitalization time, need for additional surgical procedures and medical treatment. In a recent study by Ofstead et al. where reprocessing practices of two institutions were evaluated, contamination was found in 100% of ureteroscopes after the sterilization process (67). The authors reinforce the need for frequent audits of reprocessing practices and highlight that the clinical implications of residual contamination and viable microbes found on sterilized ureteroscopes are still unknown. In that sense, no study has ever shown an inferior rate of urinary tract infection after flexible ureteroscopy with the employment of single-use devices (10). Therefore, the initial fear of cross infection with reusable scopes is not supported by existing literature and this is only a potential benefit from single-use scopes which has yet to be proven in clinical practice.

This study allowed us to perform an extensive review of published literature concerning flexible ureteroscope financial aspects. We have initially formulated questions to comprehensively evaluate all factors influencing flexible ureteroscope longevity and costs. The final formula was intended to contemplate the interests of specific parts involved in the flexible ureteroscopy industry: the surgeon who treats the patient, the manufacturer of the reusable or disposable scope, the producer of disposable instruments that sometimes is responsible for bringing the flexible scope to the operating field, and finally the institution where the ureteroscopy program takes place. So far, after extensive literature review, we may recommend using the last-generation, digital and high-performance single-use scopes in cases of high risk for ureteroscope damage. Also, when the market price of purchasing a new scope is elevated, or if ureteroscope repair price is high, migrating to single-use devices might be more cost-effective. In addition, academic centers may find a place for single-use devices in hands-on courses and residency training. Finally, no instrument is failure-proof and a back-up device, single-use or not, should always be available in case a second scope is required to finish the case.

Our study has some minuses. First, it is a review of existing previous studies and only four prospective randomized trials were considered suitable for this analysis. There is a paucity of well-designed trials concerning flexible ureteroscopy and cost-effectiveness of the procedure, thought it has large application worldwide. A second point, we have not addressed the patient perspective since it does not influence the economic aspect of the process as long as acceptable surgical outcomes are respected. Nevertheless, we must not forget that in several health care policies, the patients are the ones who are paying for all the expenditures involved. Third, we did not evaluate disposable materials as they may vary according to surgeon preference, institution policy, and ultimately would not influence the flexible ureteroscope price. Fourth, for those who do not routinely use a ureteral sheath, the finding that the ureter or ureteropelvic junction might be too tight for scope passage can only be done with the disposable scope already opened, significant increasing the cost of the unsuccessful procedure if a single-use scope is being used. Another issue not included in the analysis is that for urologists performing flexible ureterorenoscopy in an outpatient setting with no direct access to a sterilization unit, higher sterilization costs and administrative work associated with external providers might favor single-use instrument instead of the reusable. Finally, we did not study the environmental impact of using disposable devices instead of reusable scopes. Yet, a recent analysis by Davis et al. has shown that the total carbon footprint of the lifecycle assessment of the LithoVue^TM^ and reusable scope are not derisive and very similar, 4.43kg of CO2 and 4.47kg of CO2 per case, respectively (68).

## CONCLUSIONS

The cost-effectiveness of a flexible ureteroscopy program is dependent of several aspects and not scope purchase price itself. Literature lacks well-designed prospective randomized trials investigating the economic aspects of flexible ureteroscopy with single-use and reusable ureteroscopes. Disposable devices are already a reality and will progressively become the standard as manufacturing price falls. We have developed an evidence-based equation that will allow future comparisons of flexible ureteroscopy program cost-effectiveness with reusable versus single-use scopes worldwide. The main factors involved are purchase price and repair requirement-both affected by exclusivity agreement, replacement contract and insurance; scope longevity-influenced by surgical training, sterilization method, stone burden and instrumentation; reprocessing or recycling expenditure; and finally operating room expense.
